# Bioinformatic profiling of prognosis-related genes in the breast cancer immune microenvironment

**DOI:** 10.18632/aging.102373

**Published:** 2019-11-12

**Authors:** Fang Bai, Yuchun Jin, Peng Zhang, Hongliang Chen, Yipeng Fu, Mingdi Zhang, Ziyi Weng, Kejin Wu

**Affiliations:** 1 Breast Surgery, Obstetrics and Gynecology Hospital of Fudan University, Shanghai 200011, China; 2 Department of General Surgery, Shanghai International Medical Center, Shanghai 201318, China

**Keywords:** breast cancer, lymphocyte-specific kinase (LCK) metagene, estimation of STromal and Immune cells in MAlignant Tumours using Expression data (ESTIMATE), immune-related scores

## Abstract

In the microenvironment of breast cancer, immune cell infiltration is associated with an improved prognosis. To identify immune-related prognostic markers and therapeutic targets, we determined the lymphocyte-specific kinase (LCK) metagene scores of samples from breast cancer patients in The Cancer Genome Atlas. The LCK metagene score correlated highly with other immune-related scores, as well as with the clinical stage, prognosis and tumor suppressor gene mutation status (*BRCA2*, *TP53*, *PTEN*) of patients in the four breast cancer subtypes. A weighted gene co-expression network analysis was performed to detect representative genes from LCK metagene-related gene modules. In two of these modules, the levels of the co-expressed genes correlated highly with LCK metagene levels, so we conducted an enrichment analysis to discover their functions. We also identified differentially expressed genes in samples with high and low LCK metagene scores. By examining the overlapping results from these analyses, we obtained 115 genes, and found that 22 of them were independent predictors of overall survival in breast cancer patients. These genes were validated for their prognostic and diagnostic value with external data sets and paired tumor and non-tumor tissues. The genes identified herein could serve as diagnostic/prognostic markers and immune-related therapeutic targets in breast cancer.

## INTRODUCTION

In recent years, with the increasing understanding of the immune microenvironment of breast cancer tissues, immune escape has come to be considered an important marker of breast cancer development [1–4]. After tumor occurrence, tumor cells continuously interact with the immune microenvironment and gradually acquire the capacity for immune escape [5]. Both innate immunity (facilitated by macrophages and neutrophils) and adaptive immunity (facilitated by T cells and B cells) are impaired in patients with breast cancer. These impairments alter the immune microenvironment and promote the occurrence and development of tumors by (1) stimulating tumor angiogenesis, (2) altering the biological characteristics of tumors, (3) screening for tumor cells that are more suitable for survival in the host microenvironment and (4) regulating the activity of tumor stem cells. Therefore, targeted tumor immune microenvironment therapy for breast cancer has become a research hotspot [6]. However, such research has mostly been limited to preclinical experiments or clinical data-mining studies with small sample sizes [7].

Based on the expression of immune-related genes in The Cancer Genome Atlas (TCGA) database, researchers have developed a variety of immune scoring methods to investigate the interactions between tumor cells and immune cells in tumor tissues [8]. For instance, ‘Estimation of STromal and Immune cells in MAlignant Tumours using Expression data’ (ESTIMATE) is a tool that uses gene expression data to predict the purity of tumors and the presence of infiltrating stromal/immune cells in tumor tissues [9]. Previous ESTIMATE analyses have revealed that stromal/immune cell infiltration is associated with an improved prognosis in patients with various types of tumors, including prostate cancer and colorectal cancer [10, 11]. However, similar research has not been conducted in breast cancer.

Therefore, in this study, we used a series of bioinformatic tools to identify suitable immune scoring methods for different clinical subtypes of breast cancer, in order to discover diagnostic and prognostic markers of breast cancer.

## RESULTS

### Selection of the lymphocyte-specific kinase (LCK) metagene as a representative gene in the breast cancer immune microenvironment

**Figure 1:**
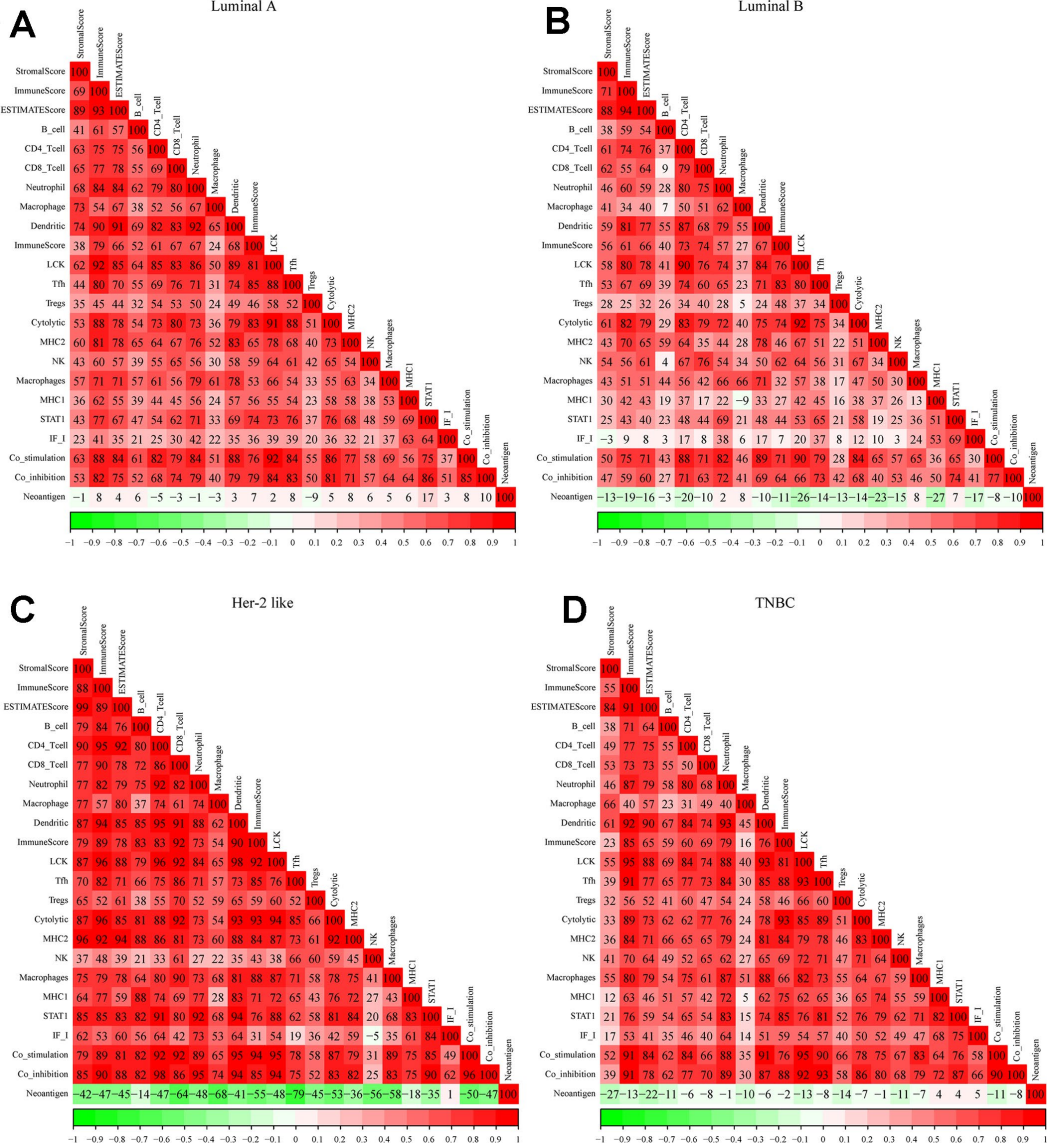
**Correlations between different immune scores in patients with different breast cancer subtypes.** (**A**) Luminal A subtype, (**B**) Luminal B subtype, (**C**) Her-2-like subtype, (**D**) TNBC subtype. Spearman correlation coefficients are color-coded to indicate positive (red) or negative (green) associations.

We obtained gene expression data from patients with different breast cancer subtypes from TCGA, and used Spearman correlation coefficients to calculate the correlations between different immune-related scores in these patients (Figure 1A–1D). With the exception of the neoantigen score, all the immune scores exhibited strong positive correlations with one another, especially in patients with the Her-2-like subtype. In all four subtypes of breast cancer, the LCK metagene score exhibited the highest mean correlation score with the other types of immune-related scores (luminal A: 77.7, luminal B: 69.4, Her-2-like: 87.7, triple-negative breast cancer [TNBC]: 76.7). Thus, we selected the LCK metagene as a representative gene in the breast cancer immune microenvironment.

**Figure 2:**
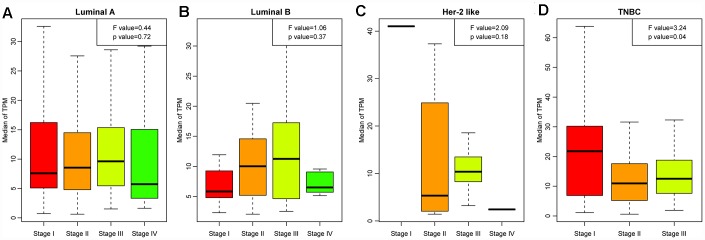
**LCK metagene scores of patients at different clinical stages.** (**A**) Luminal A subtype, (**B**) Luminal B subtype, (**C**) Her-2-like subtype, (**D**) TNBC subtype. Data are presented as the mean ± standard error of the mean (SEM).

Next, we analyzed the distribution of LCK metagene levels in patients of the four breast cancer subtypes at different clinical stages (Figure 2A–2D). LCK metagene expression was significantly upregulated in stage I in TNBC, suggesting that high LCK metagene expression may be a positive prognostic factor in TNBC.

**Figure 3:**
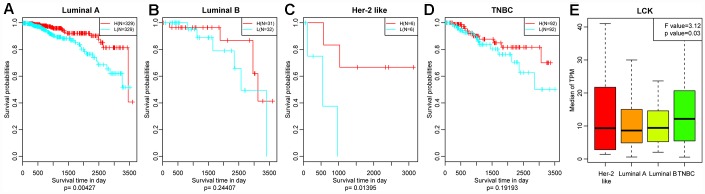
**Relationship between the LCK metagene score and prognosis.** (**A**) Luminal A subtype, (**B**) Luminal B subtype, (**C**) Her-2-like subtype, (**D**) TNBC subtype. Data were analyzed in KM plotter. H: high LCK metagene score; L: low LCK metagene score. The log-rank p values are shown. (**E**) LCK metagene scores of patients with different breast cancer subtypes. Data are presented as the mean ± SEM.

We then divided the patients of each breast cancer subtype into two groups based on the median LCK metagene level, and assessed the prognostic differences between patients with high and low LCK metagene levels (Figure 3A–3D). The prognosis of the high expression group was better than that of the low expression group in all four subtypes. We also observed significant differences in LCK metagene expression among the four subtypes (Figure 3E). The median LCK metagene score was significantly higher in the TNBC group than in the other groups, suggesting that LCK metagene expression can be used as a prognostic marker in breast cancer.

**Figure 4:**
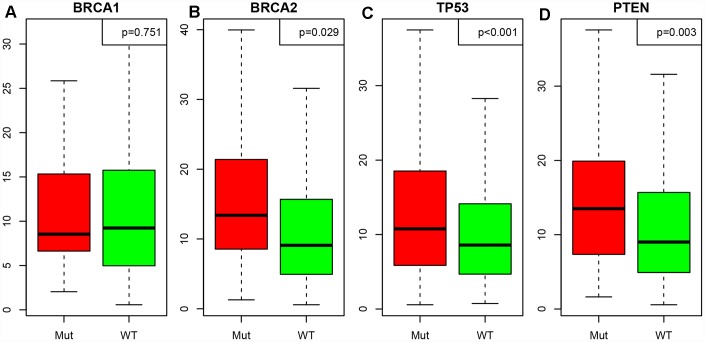
**Correlation between the LCK metagene score and gene mutations.** (**A**) *BRCA1*, (**B**) *BRCA2*, (**C**) *TP53*, (**D**) *PTEN*. Mut: mutant; WT: wild-type. Data are presented as the mean ± SEM.

Next, we downloaded single-nucleotide polymorphism data on *BRCA1*, *BRCA2*, *TP53* and *PTEN* [12], and divided patients into mutant and wild-type groups. We then determined the LCK metagene expression in each of these groups (Figure 4A–4D). LCK metagene expression was significantly greater in the *BRCA2*, *TP53* and *PTEN* mutant groups than in their wild-type counterparts, but did not differ significantly between the *BRCA1* mutant and wild-type groups.

Thus, the LCK metagene can be regarded as a representative gene in the immune microenvironment of breast cancer, and can be explored as a marker for the development of drugs to treat breast cancer.

### Screening of representative genes in LCK metagene-related gene modules

We then performed a hierarchical clustering analysis (Supplementary Figure 1A). Samples with a distance >80,000 were screened as outliers, and 1146 samples were ultimately obtained. Then, weighted gene co-expression network analysis (WGCNA) was used to construct a weighted co-expression network, and a β value of 8 was used to ensure a scale-free network (Supplementary Figure 1B and 1C). This analysis yielded 34 modules (Supplementary Figure 1D). Gene sets that could not be aggregated into other modules are shown as grey modules. In total, 7530 transcripts were allocated to 34 co-expression modules, and the transcripts of each module are shown in Supplementary Table 1. The correlations between the eigenvectors of these 34 modules and the LCK metagene score were calculated (Supplementary Figure 2). The LCK metagene score had a very high correlation with the red module (R=0.97), followed by the magenta module (R=0.64).

**Figure 5:**
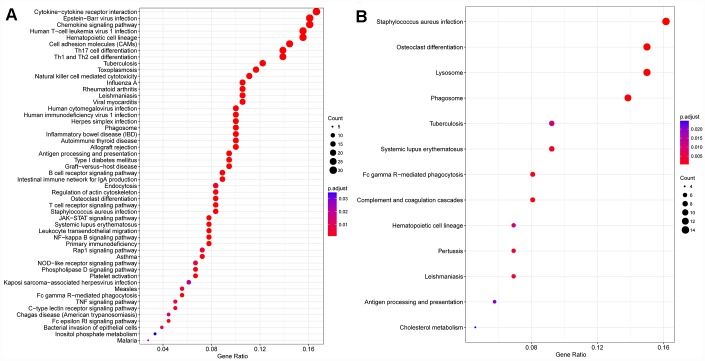
**Gene KEGG pathway enrichment analysis.** (**A**) Red module, (**B**) magenta module.

Next, we selected the red and magenta modules for Kyoto Encyclopedia of Genes and Genomes (KEGG) enrichment analyses (see “module all kegg enrich.txt”). The red module was enriched in 52 pathways that were associated with various aspects of immunity, including the hematopoietic cell lineage, Th1 and Th2 cell differentiation and Th17 cell differentiation (Figure 5A). The magenta module was enriched in 13 pathways (Figure 5B), which were mainly associated with lysosomes and phagosomes.

We then analyzed the correlations between the genes of these two modules and determined the correlation distribution of these genes in breast cancer patients from TCGA (Supplementary Figure 3). The correlation coefficients were bimodally distributed. We selected 162 genes with maximum correlation coefficients >0.79 between the two modules (see “Module.gene.cor.txt”), reasoning that these genes could be associated with members of the LCK metagene.

### Identification of differentially expressed genes (DEGs) in the high and low LCK metagene expression groups

**Figure 6:**
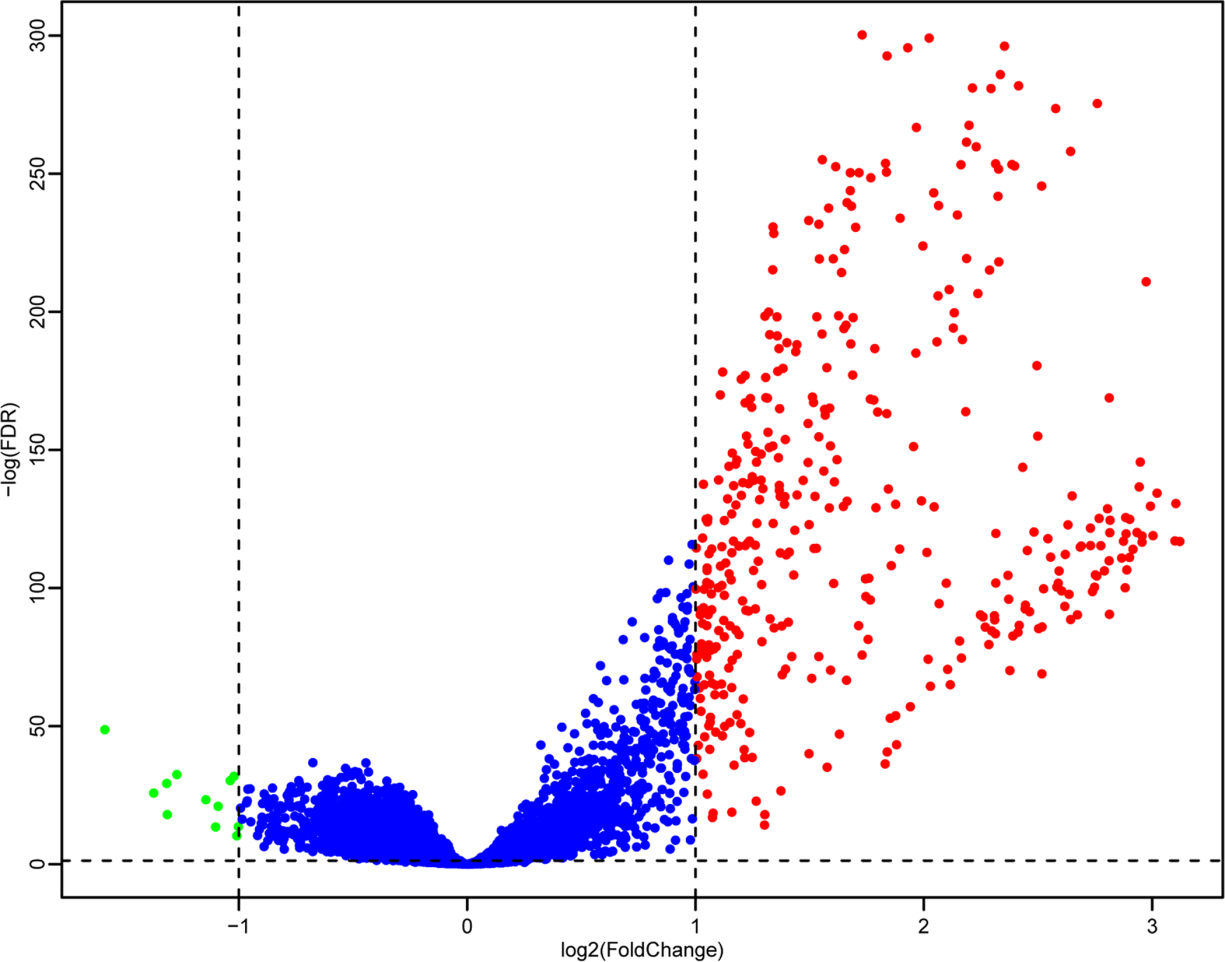
**Volcano maps of DEGs.** Red represents genes that were upregulated in patients with high LCK metagene scores, while green represents genes that were upregulated in patients with low LCK metagene scores.

Next, we used the DESeq2 function in the R software package [13] to analyze the genetic differences between the high and low LCK metagene expression groups, and obtained 403 DEGs. The volcano plot is shown in Figure 6. There were significantly more upregulated genes than downregulated genes in the high LCK metagene expression group.

### Exploration of prognostic markers related to the immune microenvironment of breast cancer

**Figure 7:**
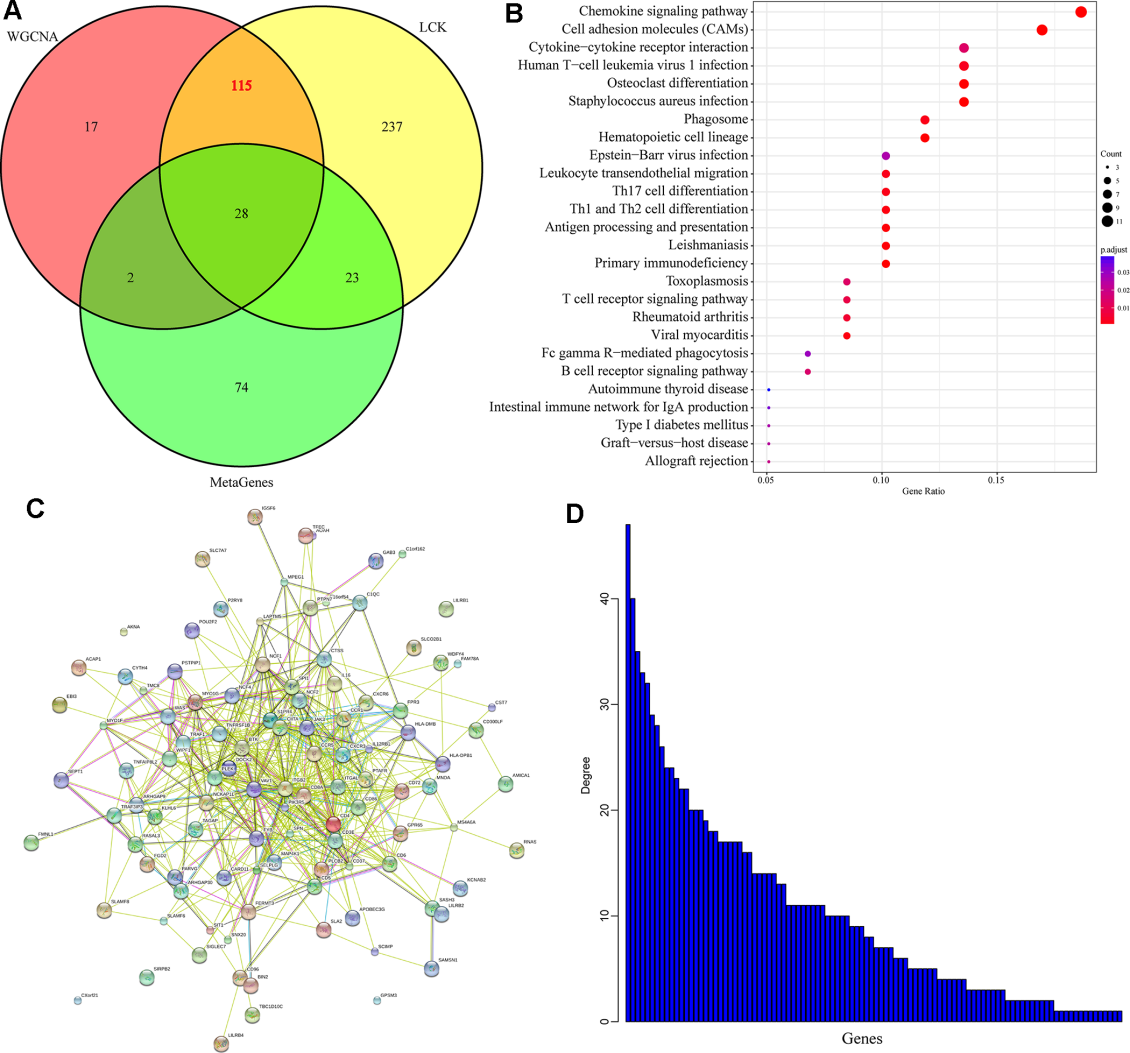
**Prognostic markers related to the immune microenvironment of breast cancer.** (**A**) Co-expressed genes that significantly correlated with gene members of the LCK metagene in terms of their mRNA levels. (**B**) KEGG enrichment analysis of the 115 genes. (**C**) Protein interaction networks of the 115 genes. (**D**) The degree distribution of nodes in the network.

We then integrated the 162 genes from the two most relevant modules of the LCK metagene and the 403 DEGs between the high and low LCK metagene expression groups. From this integration, 143 genes were selected, but 28 genes from the known immune-related metagenes were excluded, resulting in 115 genes (Figure 7A) (last.genes.deg.txt). We used the R software package clusterProfiler for KEGG enrichment analysis of these genes, employing a false discovery rate <0.05 as the threshold value (Figure 7B) (last.genes.deg.kegg.txt). Forty-two genes were enriched in 26 pathways, most of which were associated with immune diseases.

We used the R software package STRINGdb to analyze the protein network interactions of these 115 genes. When the genes were mapped into the STRING database, a relationship network containing 526 edges and 102 nodes was obtained (Figure 7C). We analyzed the degree distribution of the nodes in the network (Figure 7D), and found that the degree of each node was high (10.3, on average), suggesting that the genes were closely related.

Next, we performed a univariate survival analysis to determine the relationship between the expression of these 115 genes and prognosis in breast cancer patients from TCGA. Using a p value <0.05 as a threshold, we selected 28 genes for which high expression was associated with a good prognosis. In order to exclude the influence of the clinical stage, we subsequently included the clinical stage as a covariable in the analysis, and ultimately obtained 22 independent prognostic factors, as shown in Table 1. We then used the online analysis tool g:profiler to analyze the Gene Ontology (GO) Terms of these 22 genes (Table 2) [14]. Thirteen genes were enriched for eight GO Terms associated with immunity, while the remaining nine genes (*ACAP1*, *SEPT1*, *MAP4K1*, *TRAC*, *CXCR6*, *TRBC2*, *TRAF1*, *PTPN7* and *TRBV28*) were not enriched for any GO Terms.

**Table 1:** Genes with prognostic value.

**Genes**	**Symbol**	**P value**	**HR**	**Low 95%CI**	**High 95%CI**
ENSG00000015285	WAS	0.040077	0.984523	0.969971	0.999294
ENSG00000072818	ACAP1	0.041169	0.96313	0.929017	0.998495
ENSG00000137078	SIT1	0.046466	0.977609	0.956059	0.999646
ENSG00000180096	SEPT1	0.02709	0.967847	0.9402	0.996306
ENSG00000104814	MAP4K1	0.045689	0.97372	0.948614	0.99949
ENSG00000186810	CXCR3	0.022463	0.97858	0.960553	0.996945
ENSG00000277734	TRAC	0.027988	0.996204	0.99283	0.999589
ENSG00000153563	CD8A	0.021632	0.984938	0.972265	0.997777
ENSG00000172215	CXCR6	0.025982	0.950541	0.909031	0.993947
ENSG00000211772	TRBC2	0.013298	0.99403	0.989328	0.998753
ENSG00000056558	TRAF1	0.042017	0.972292	0.946311	0.998986
ENSG00000143851	PTPN7	0.038225	0.967811	0.938323	0.998226
ENSG00000198851	CD3E	0.018692	0.991008	0.983575	0.998497
ENSG00000175463	TBC1D10C	0.023773	0.963748	0.933385	0.995099
ENSG00000239713	APOBEC3G	0.012421	0.966283	0.940647	0.992619
ENSG00000160593	JAML	0.034375	0.946377	0.899269	0.995953
ENSG00000211753	TRBV28	0.022437	0.989539	0.980646	0.998514
ENSG00000278030	TRBV7-9	0.048429	0.975	0.950792	0.999825
ENSG00000223865	HLA-DPB1	0.011418	0.998766	0.997812	0.999722
ENSG00000125910	S1PR4	0.039385	0.9641	0.931142	0.998224
ENSG00000013725	CD6	0.036304	0.971208	0.945004	0.998138
ENSG00000077984	CST7	0.010964	0.988294	0.979369	0.997301

HR: hazard ratio; CI: confidence interval.

**Table 2:** GO enrichment of 22 immune-related genes.

**GO.ID**	**Description**	**P value**	**FDR**	**Genes**
GO:0050852	T cell receptor signaling pathway	1.79E-02	1.79E-02	WAS, CD3E, HLA-DPB1, TRBV7-9,
GO:0002376	immune system process	3.91E-03	3.91E-03	CD6, WAS, CST7, S1PR4, SIT1, CD8A, JAML, TBC1D10C, CXCR3, CD3E, HLA-DPB1, APOBEC3G, TRBV7-9
GO:0006955	immune response	7.97E-04	7.97E-04	CD8A, JAML, TBC1D10C, CD3E, HLA-DPB1, APOBEC3G, TRBV7-9
GO:0002682	regulation of immune system process	1.96E-04	1.96E-04	CD6, WAS, SIT1, CD8A, JAML, TBC1D10C, CXCR3, CD3E, HLA-DPB1, APOBEC3G, TRBV7-9
GO:0045321	leukocyte activation	4.93E-02	4.93E-02	CD6, WAS, SIT1, CD8A, JAML, TBC1D10C, CD3E, HLA-DPB1
GO:0046649	lymphocyte activation	6.51E-04	6.51E-04	CD6, WAS, SIT1, CD8A, JAML, TBC1D10C, CD3E, HLA-DPB1
GO:0042110	T cell activation	5.91E-04	5.91E-04	CD6, WAS, SIT1, CD8A, JAML, CD3E, HLA-DPB1
GO:0042101	T cell receptor complex	2.15E-03	2.15E-03	CD6, CD8A, CD3E

FDR: false discovery rate.

Breast cancer patient samples from TCGA were then divided into two groups based on the median levels of the 22 prognosis-related genes. The prognostic differences between the high and low expression groups for each of these 22 genes were analyzed. As shown in Supplementary Figure 4, high expression of these 22 genes was associated with a significantly better prognosis than low expression. Thus, these 22 genes could be used as new prognostic markers or therapeutic targets related to the immune microenvironment of breast cancer.

### Association of the 22 immune-related prognostic genes with LCK metagene members

To examine the relationship between the LCK metagene and the 22 newly discovered prognostic genes related to the immune microenvironment, we extracted the expression profiles of the 42 gene members of the LCK metagene and the 22 genes related to immune microenvironment from breast cancer patients in TCGA. A clustering analysis was performed on the expression profiles, and the Euclidean distance was adopted (Supplementary Figure 5A). The expression patterns of the 22 immune-related prognostic genes were very similar to those of the 42 members of the LCK metagene. The samples formed three obvious clusters, so we further analyzed the expression distribution of the three groups of samples (Supplementary Figure 5B). The expression order was Cluster2>Cluster1>Cluster3. Then, we analyzed the prognostic differences among the three groups, and found a significant difference in the five-year survival rate, as follows: Cluster2>Cluster1> Cluster3 (Supplementary Figure 5C).

### External data validation

**Figure 8:**
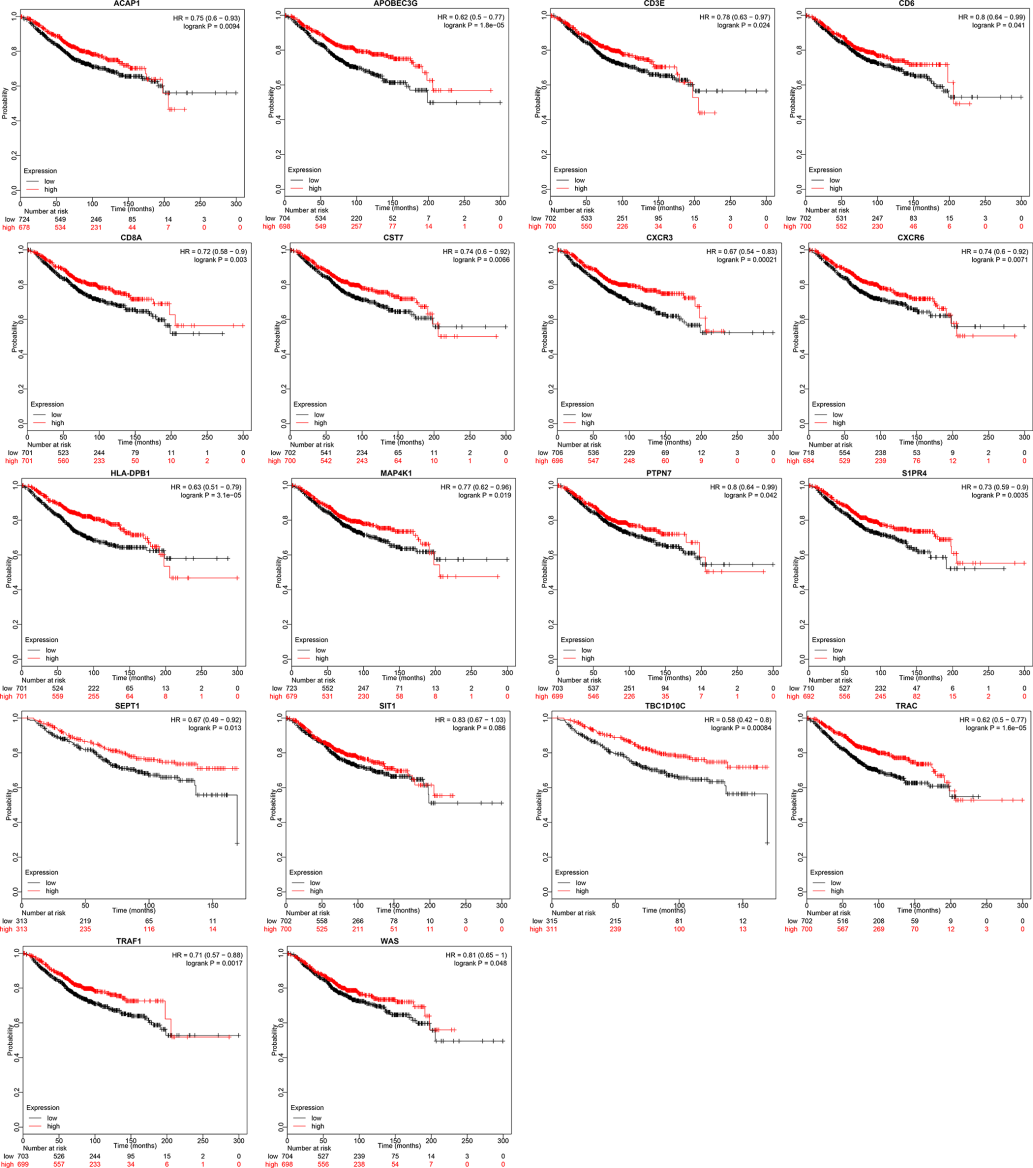
**Relationship between immune microenvironment-related genes and breast cancer patient prognosis.** Data were analyzed with KM plotter. Probabilities indicate overall survival; HR: hazard ratio.

To verify the prognostic value of these 22 genes, we used the breast cancer dataset from the online tool Kaplan-Meier (KM) plotter [15] (http://kmplot.com) to analyze the relationship between the expression of these genes and the overall survival of breast cancer patients. We assessed the 18 genes with available data on the KM plotter platform, and divided the samples into high and low expression groups according to the median mRNA level of each gene. The KM curves of the 18 genes are shown in Figure 8. High expression of these 18 genes was associated with a good prognosis, consistent with our earlier analysis results.

### Specimen verification results

We then analyzed the expression of the 22 prognosis-related genes in tumor and non-tumor tissues from five breast cancer patients. The fold-changes in these 22 genes between tumor and non-tumor tissues (T/N) are shown in Supplementary Figure 6. In most cases, the fold-change (T/N) was less than 0.05, indicating that the gene was downregulated in breast cancer tumors (all data are available upon request).

## DISCUSSION

In recent years, breast cancer treatment has included surgery, chemotherapy, endocrine therapy, targeted therapy and radiotherapy. However, traditional therapies have failed to save some refractory patients [16–18]. In this context, increasing attention has been paid to the tumor microenvironment and tumor immunity [19]. Stimulating the immune system and enhancing the anti-tumor function of the tumor microenvironment may be a novel way to kill tumor cells [20]. Therefore, screening genes related to the immune environment of breast cancer is an important step towards predicting the prognosis of patients and identifying new therapeutic targets. In this study, we used the database of TCGA to search for immune microenvironmental markers associated with the overall survival of breast cancer patients. We found that the expression of 22 genes correlated significantly with overall survival, and verified these results in KM plotter.

We first demonstrated that the LCK metagene score correlated highly with various other immune-related scores, along with the tumor clinical stage, the prognosis and the mutation status of multiple tumor suppressor genes (*BRCA2*, *TP53* and PTEN) in patients with the four subtypes of breast cancer (luminal A, luminal B, Her-2-like and TNBC). *BRCA1* and *BRCA2* have been described as “breast cancer susceptibility genes,” so failure to properly repair mutations in these genes increases the risk for breast cancer [21]. Our data indicated that *BRCA2* mutations, but not *BRCA1* mutations, correlated highly with LCK metagene expression. It is worth noting that BRCA1 and BRCA2 are unrelated proteins that were discovered separately [22]. Until now, little has been reported about the relationship between the LCK metagene and BRCA1/2. Based on the current evidence, *BRCA1* and LCK metagene expression may be independent markers of breast cancer.

Secondly, taking the gene members of the LCK metagene as the research object, we used a WGCNA to detect representative genes from the relevant gene modules of the LCK metagene. We also analyzed the DEGs between samples with high and low LCK metagene scores to identify co-expressed genes that correlated significantly with members of the LCK metagene in terms of their mRNA levels. Then, we assessed the overlap between these gene sets, and performed a survival analysis to determine which of these co-expressed genes were significantly associated with the prognosis of breast cancer patients. We explored the functions of these genes through an enrichment analysis, and further verified our findings with external data sets. We ultimately found 22 potential immune-related diagnostic and prognostic markers: *WAS*, *ACAP1*, *SIT1*, *SEPT1*, *MAP4K1*, *CXCR3*, *TRAC*, *CD8A*, *CXCR6*, *TRBC2*, *TRAF1*, *PTPN7*, *CD3E*, *TBC1D10C*, *APOBEC3G*, *JAML*, *TRBV28*, *TRBV7-9*, *HLA-DPB1*, *S1PR4*, *CD6* and *CST7*.

The CD8 antigen is a cell-surface glycoprotein found on most cytotoxic T lymphocytes, and facilitates efficient cell-cell interactions within the immune system. *CD8A* encodes the CD8 alpha chain, and was one of the 22 immune-related genes identified in this study. Cytotoxic T lymphocytes can recognize and eliminate infected cells and tumor cells. CD8A homodimers on the surface of natural killer cells enable these cells to conjugate with and lyse multiple target cells, thus promoting survival [23]. Higher CD8A expression has been associated with a better prognosis in breast cancer patients [24]. Thus, therapeutically inducing CD8A could enhance the function of cytotoxic T lymphocytes, enabling them to kill more cancer cells.

*CXCR3*, another one of the 22 genes we identified, encodes a G protein-coupled receptor that is selective for three chemokines: CXCL9, CXCL10 and CXCL11. Binding of chemokines to CXCR3 induces cellular responses that are involved in leukocyte trafficking, most notably integrin activation, cytoskeletal changes and chemotactic migration [25]. In mice, CXCR3 deficiency was reported to promote the development of breast cancer by stimulating the M2 polarization of macrophages [26]. Higher CXCR3 expression was found to predict favorable outcomes in breast cancer patients treated with tamoxifen [27].

Another immune-related prognostic gene detected in this study was *CXCR6*. This gene is expressed by activated natural killer cells. In a previous study, irradiation was reported to induce CXCL16 chemokine expression in cancer cells and to enhance the migration of CXCR6+ natural killer cells to breast cancer cells for their destruction [28].

Among the 22 genes we identified, 15 (*ACAP1*, *MAP4K1*, *CXCR3*, *TRAC*, *CD8A*, *CXCR6*, *TRBC2*, *TRAF1*, *CD3E*, *APOBEC3G*, *TRBV28*, *TRBV7-9*, *HLA-DPB1*, *CD6* and *CST7*) have previously been reported to be involved in the occurrence, development, malignant transformation and pathology of breast cancer, and to be associated with the survival and prognosis of patients [29–33]. This emphasizes the reliability and accuracy of our biological information mining results based on TCGA database screening and KM plotter database verification. The same gene expression trends were detected in matched tumor and non-tumor tissues, further validating our results. However, the remaining seven genes (*S1PR4*, *SIT1*, *AML*, *PTPN7*, *WAS*, *TBC1D10C* and *SEPT1*) have not been reported to be associated with breast cancer in experimental or clinical studies. Among these genes, *SIT1* and *JAML* are of the greatest interest to us. SIT1 regulates the proliferation, activation and survival of memory T cells, thus affecting the generation of regulatory T cells, the immune escape of tumors and the resistance of tumors to immunotherapy [34]. JAML is involved in the proliferation and survival of T cells, as well as the production and release of cytokines and growth factors; thus, JAML regulates the sensitivity of tumor cells to relevant vaccines [35].

This study had several limitations. Firstly, our prognostic analyses of genes were based on overall survival, but information on relapse-free survival was lacking. Secondly, although most of the genes we identified could be verified in external databases and patient samples, the KM plotter platform could only verify 18 genes. Thirdly, the data from TCGA came from tissues; thus, although we identified 22 genes that could be therapeutic targets in breast cancer, we could not determine which cells highly expressed these genes. However, using an online tool (http://biogps.org), we were able to determine which types of cells typically express certain genes. For instance, CD19+ B cells highly express *HLA-DBP1*, and CD8+ T cells highly express *CD8A*. Single-cell sequencing can now be used to explore which cells highly express specific genes [36]; thus, our data could provide a reference for future single-cell sequencing analyses.

Although many studies have described the correlation between gene expression and survival in breast cancer patients, the results of most of these studies have been verified in animal tumor models, *in vitro* cell models or small numbers of human samples. The infiltration of immune cells (regulatory T cells, M2 tumor-related macrophages and CD20+ B cells) in the microenvironment of breast cancer is an important predictor of prognosis [37–39]. Retrospective studies have indicated that higher expression of immune checkpoint molecules (PD-L1, PD-1, CTLA-4 and LAG3) is associated with a higher survival rate in TNBC patients [40]. However, the complexity of the breast cancer microenvironment requires more comprehensive analyses in larger study populations. Fortunately, the rapid development of whole-genome sequencing and the development of high-throughput tumor databases such as TCGA have made it possible to analyze ‘big data’ from large-scale breast cancer populations. Furthermore, the ESTIMATE method can be applied to detect the infiltration of stromal and immune cells into tumor samples based on gene expression data [10]. In this study, we focused on immune microenvironment-related genes that were involved in the occurrence, development and malignant transformation of breast cancer and the overall survival of patients. Our results have helped to decode the complex microenvironment of breast cancer, and can be used as a source of potential immune-related diagnostic/prognostic markers or therapeutic targets for breast cancer.

## MATERIALS AND METHODS

### Data sources and pre-processing

Counts data (see TCGA-BRCA counts.txt; all materials are available upon request), single-nucleotide polymorphism data (see TCGA.mutect.somatic.maf) and clinical follow-up information (see Merge clinilcal.txt) were downloaded from the database of TCGA. RNA-Seq data (reads per kilobase million) were downloaded from TCGA and converted into transcripts per kilobase million (TPM) expression profiles (see Merge TCGA-BRCA TPM.txt). Thirteen metagenes (see ImmuneScore.genes.ids.txt) corresponded to various immune cell types and reflected the corresponding immune functions [41]. The median mRNA levels of these immune metagenes were used for scoring (see meta.score.txt). The scores of immune cells in samples (six categories) were calculated through the Tumor Immune Estimation Resource (https://cistrome. shinyapps.io/timer/) (immu.score.txt) [42]. The immune and stromal scores of samples were calculated with the ESTIMATE function of the R software package (est.score.txt). The immune neoantigen score was calculated by a previously reported method [43] (Neoantigen.txt).

### Screening representative genes in the breast cancer immune microenvironment

We used Spearman correlation coefficients to calculate the correlations between different immune-related scores in different breast cancer subtypes. Based on the results, the LCK metagene was selected as a representative gene in the breast cancer immune microenvironment. Next, we analyzed the relationship between LCK metagene expression and clinical stage. We also classified samples into high and low expression groups according to the median mRNA level of the LCK metagene, and performed KM analysis to determine the prognostic differences between the groups. In addition, we analyzed the relationship between LCK metagene expression and *BRCA1*, *BRCA2*, *TP53* and *PTEN* mutations.

### Analysis of LCK metagene-related modules by WGCNA

We used the R software package WGCNA [44] to construct a weighted co-expression network. A dynamic shearing method was used to generate gene modules, and a cluster analysis was carried out on the modules. Closely spaced modules were merged into a new module, and the height, deepSplit and minModuleSize were set at 0.25, 2 and 30, respectively. We used the R software package clusterProfiler for KEGG enrichment analysis (false discovery rate <0.05) of genes from two modules of interest. We then explored the genes associated with the LCK metagene, and calculated the correlations in gene expression between the two modules of interest. We selected genes with the maximum correlation coefficients from the modules.

### Screening immune microenvironment genes related to prognosis

According to their LCK metagene scores, samples were divided into two groups: the high LCK group and the low LCK group. Then, the DESeq2 function in the R software package [14] was used to analyze the genetic differences between the two groups of samples. First, we extracted 15,268 transcripts with TPM values >1 in more than 75% of the samples and a median absolute deviation greater than the median. Then, we screened the DEGs to obtain those with a false discovery rate <0.05 and a |log2(Foldchange)|>1), and used the R software package clusterProfiler for KEGG enrichment analysis of these genes. To identify genes with prognostic value in the immune microenvironment, we performed a univariate survival analysis, as shown in “lst.cox.txt”. We also used the online analysis tool g:profiler to analyze the GO Terms of these genes. Then, we used the online tool KM plotter to analyze the relationship between the expression of these genes and the overall survival of breast cancer patients.

### Specimen verification

We collected five paired tumor and non-tumor samples from breast cancer patients. Then, we used a PrimeScript™ RT reagent Kit (Cat#RR037A, Takara, Japan) to extract RNA from these samples and to reverse-transcribe the RNA to cDNA. Gene expression was detected by quantitative polymerase chain reaction experiments (TB Green™ Premix Ex Taq™ II, Cat#RR820A, Takara, Japan). Transcription products were quantified relative to beta-actin. The tumor and non-tumor tissues were collected with informed consent. This study was approved by the Ethics Committee of the Obstetrics and Gynecology Hospital of Fudan University.

### Ethical statement

Samples from patients in this study were used with approval from the Ethics Committee of the Obstetrics and Gynecology Hospital of Fudan University and with consent from all the patients.

## Supplementary Material

Supplementary Figures

Supplementary Table 1
