# Supplementary material for: Bioinformatic profiling of prognosis-related genes in the breast cancer immune microenvironment
Source: Aging (Albany NY). 2019 Nov 12;11(21):9328–47. doi: 10.18632/aging.102373 (PMC6874454; doi:10.18632/aging.102373)
Supplement: Supplementary Figures [file aging-11-102373-s002.pdf]

SUPPLEMENTARY FIGURES

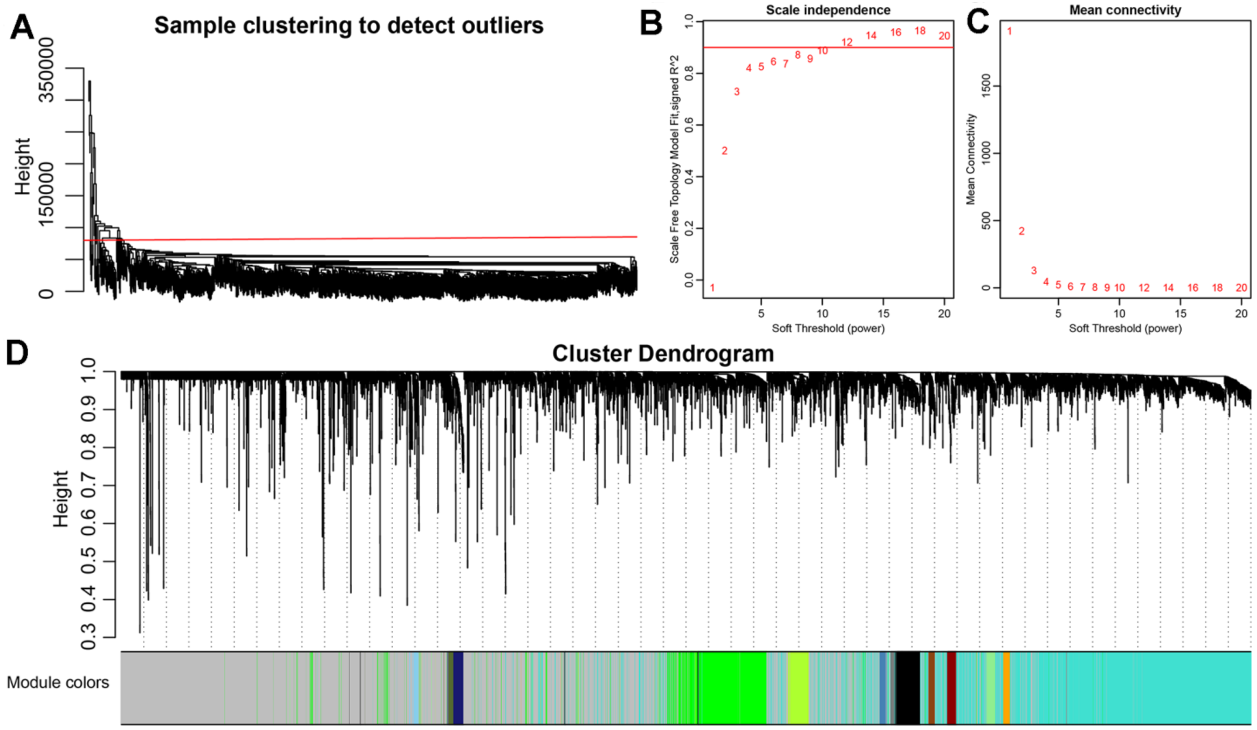

Supplementary Figure 1. LCK metagenes-related gene modules mined through WGCNA. (**A**) Sample clustering analysis. (**B**, **C**) Analysis of network topology under various soft-thresholding powers. (**D**) Gene dendrogram and module colors.

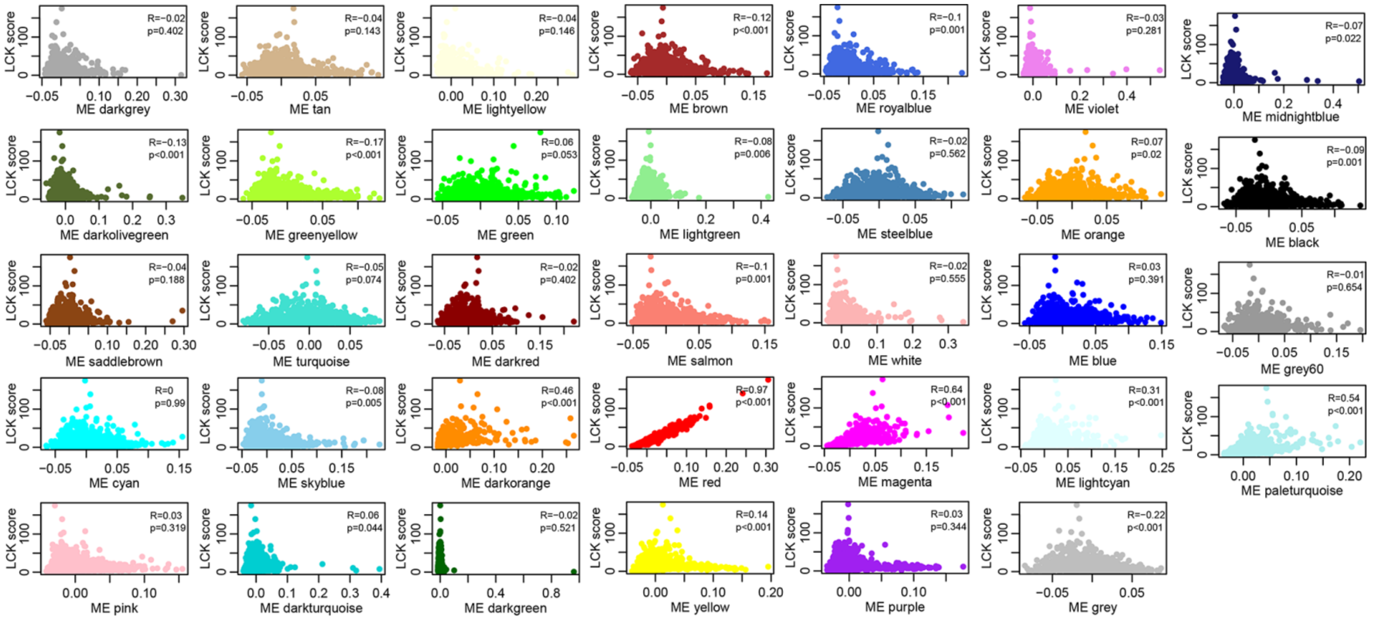

Supplementary Figure 2. Correlation between eigenvectors of 34 gene modules and LCK metagenes.

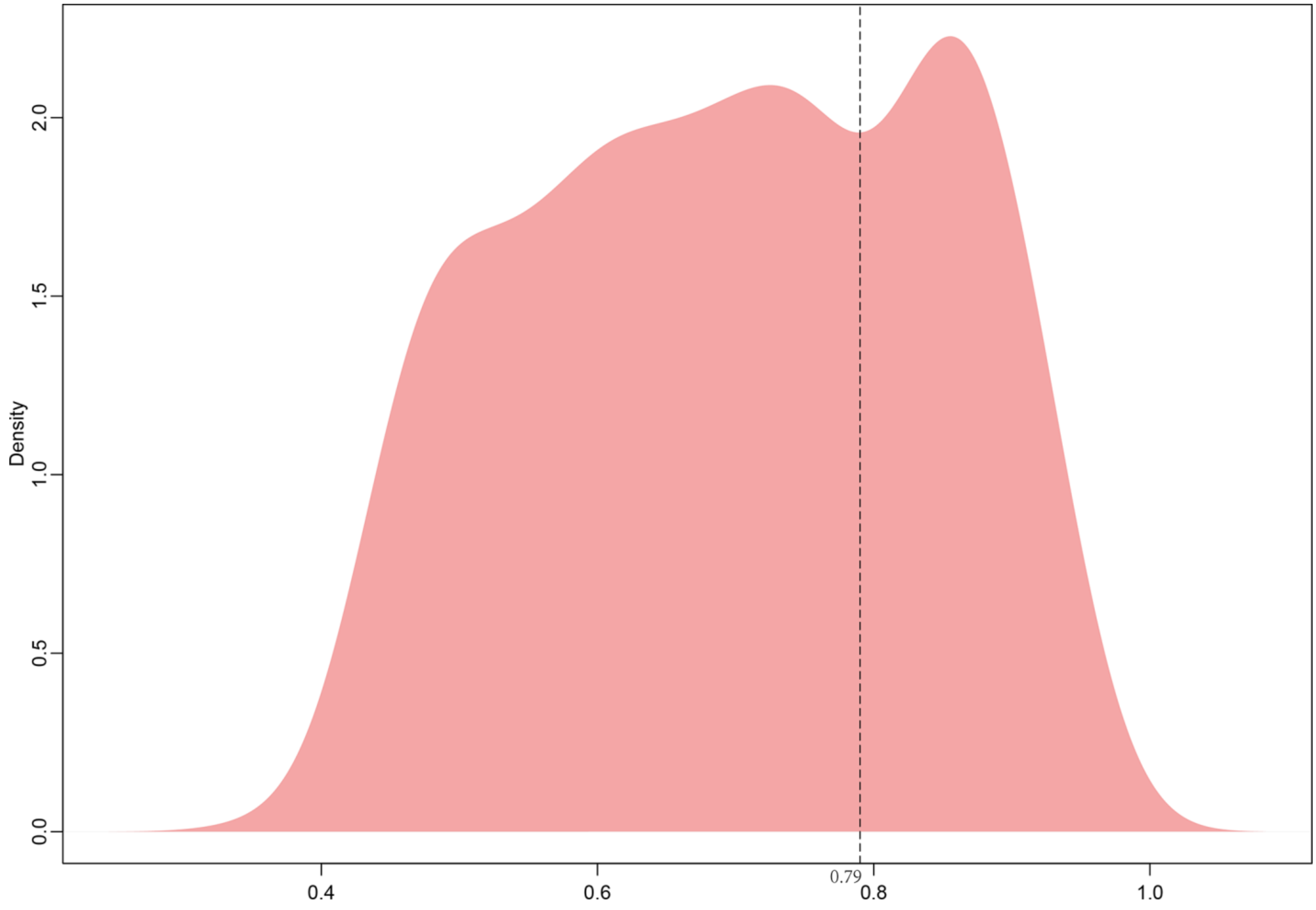

Supplementary Figure 3. Screening of module-related genes.

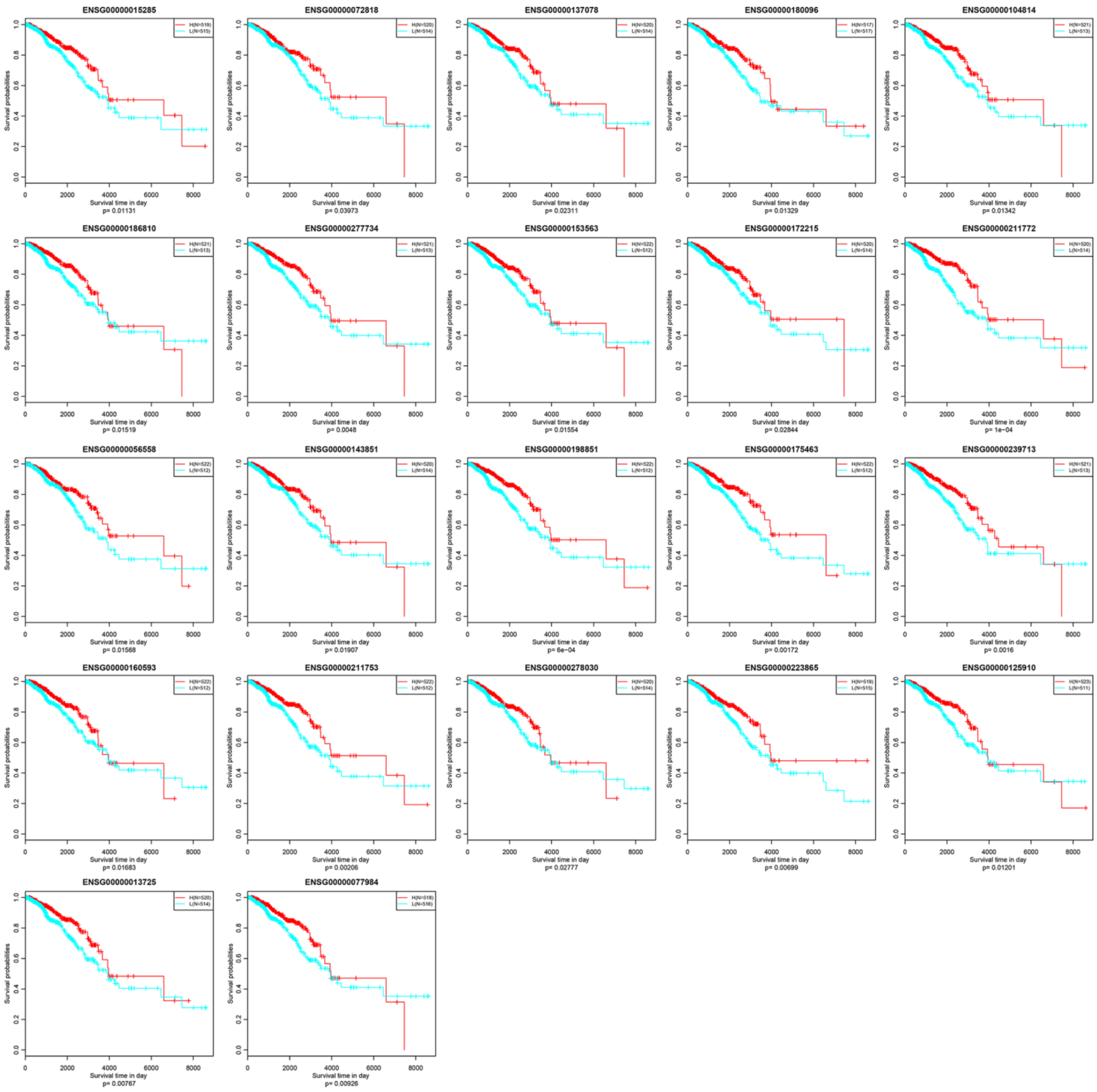

Supplementary Figure 4. Relationship between 22 immune microenvironment-related genes and prognosis. Kaplan-Meier survival curve between high expression and Low expression group of breast cancer patients.

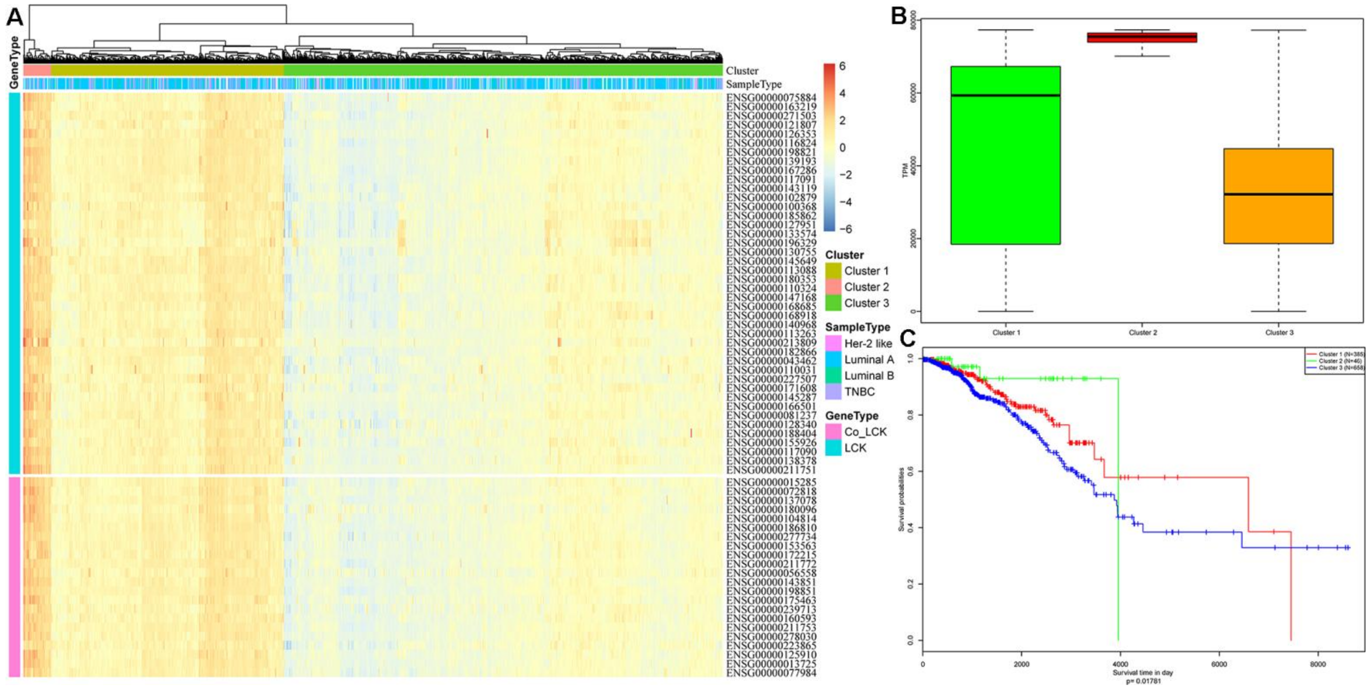

Supplementary Figure 5. Correlations of 22 immune microenvironment-related prognostic genes and LCK metagenes. (**A**) Expression profiles of 22 immune microenvironment-related prognostic genes and 42 gene members in LCK metagenes. (**B**) LCK metagenes scores in the three clusters. (**C**) Kaplan-Meier survival curves for the three clusters.

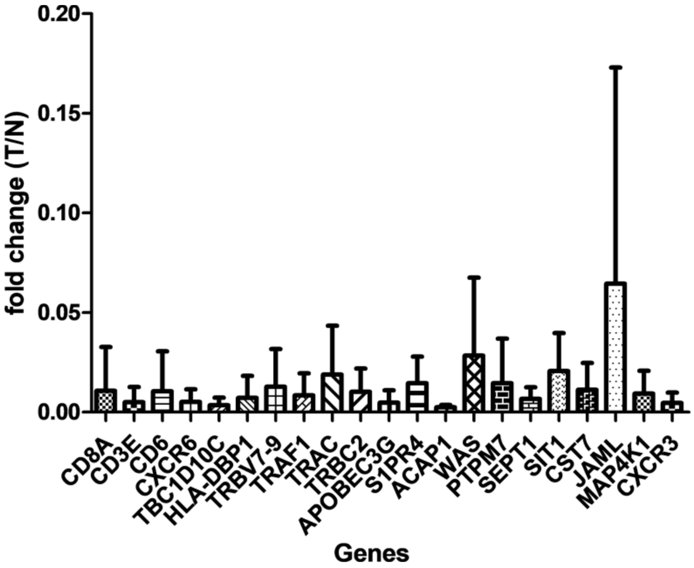

Supplementary Figure 6. Fold change of 22 genes. Data are represented as mean +/- SD.
