# Supplementary material for: Bioinformatic profiling of prognosis-related genes in the breast cancer immune microenvironment
Source: Aging (Albany NY). 2019 Nov 12;11(21):9328–47. doi: 10.18632/aging.102373 (PMC6874454; doi:10.18632/aging.102373)
Supplement: Supplementary Table 1 [file aging-11-102373-s001.pdf]

SUPPLEMENTARY TABLE

Supplementary Table 1. The number of genes corresponding to each module.

Module	Number
black	174
blue	848
brown	755
cyan	106
darkgreen	54
darkgrey	47
darkolivegreen	32
darkorange	43
darkred	56
darkturquoise	51
green	674
greenyellow	138
grey	7437
grey60	63
lightcyan	64
lightgreen	62
lightyellow	60
magenta	155
midnightblue	66
orange	45
paleturquoise	39
pink	169
purple	139
red	355
royalblue	59
saddlebrown	41
salmon	127
skyblue	41
steelblue	41
tan	127
turquoise	2076
violet	37
white	42
yellow	744
